# Quantifying Species Diversity with a DNA Barcoding-Based Method: Tibetan Moth Species (Noctuidae) on the Qinghai-Tibetan Plateau

**DOI:** 10.1371/journal.pone.0064428

**Published:** 2013-05-31

**Authors:** Qian Jin, Huilin Han, XiMin Hu, XinHai Li, ChaoDong Zhu, Simon Y. W. Ho, Robert D. Ward, Ai-bing Zhang

**Affiliations:** 1 College of Life Sciences, Capital Normal University, Beijing, People's Republic of China; 2 School of Forestry, Experiment Center, Northeast Forestry University, Haerbin, People's Republic of China; 3 Key Laboratory of Zoological Systematics and Evolution, Institute of Zoology, Chinese Academy of Sciences, Beijing, People's Republic of China; 4 School of Biological Sciences, University Of Sydney, Sydney, Australia; 5 Wealth from Oceans Flagship, CSIRO Marine and Atmospheric Research, Hobart, Tasmania, Australia; University of Guelph, Canada

## Abstract

With the ongoing loss of biodiversity, there is a great need for fast and effective ways to assess species richness and diversity: DNA barcoding provides a powerful new tool for this. We investigated this approach by focusing on the Tibetan plateau, which is one of the world's top biodiversity hotspots. There have been few studies of its invertebrates, although they constitute the vast majority of the region's diversity. Here we investigated species diversity of the lepidopteran family Noctuidae, across different environmental gradients, using measurements based on traditional morphology as well as on DNA barcoding. The *COI* barcode showed an average interspecific K2P distance of 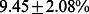, which is about four times larger than the mean intraspecific distance (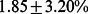). Using six diversity indices, we did not detect any significant differences in estimated species diversity between measurements based on traditional morphology and on DNA barcoding. Furthermore, we found strong positive correlations between them, indicating that barcode-based measures of species diversity can serve as a good surrogate for morphology-based measures in most situations tested. Eastern communities were found to have significantly higher diversity than Western ones. Among 22 environmental factors tested, we found that three (precipitation of driest month, precipitation of driest quarter, and precipitation of coldest quarter) were significantly correlated with species diversity. Our results indicate that these factors could be the key ecological factors influencing the species diversity of the lepidopteran family Noctuidae on the Tibetan plateau.

## Introduction

The quantification of species diversity, and understanding the processes that drive variation in diversity across space and time, form the basis of some of the most fundamental questions in ecology and evolutionary biology. Measures of species diversity are often regarded as indicators of ecosystem health and function [1]. An extensive range of indices and models for measuring diversity have been devised, and these have been surrounded by considerable debate [1]–[8]. Traditionally, these have been based on morphology-based species identification for most metazoan groups. However, morphological identification alone is not always feasible, especially for a community with a largely undescribed biodiversity [9]–[10]. DNA barcoding has gained an important role as part of efforts to develop a global inventory of biodiversity [11]–[26], but it has also been accompanied by various reservations [27]–[35]. On 14 March 2013, there were 2,012,391 barcode records from 172,280 species in the Barcode of Life Data Systems (BOLD) (www.barcodinglife.org). About 74% of the records and 82% of the species are from arthropods. DNA barcoding studies can be generally classified into theoretical and empirical studies. Theoretical studies focus on the methodology of DNA barcoding, such as the precision of species assignment or identification with a variety of methods, including classical phylogenetic approaches [16], [17], [36], [37], pure statistical approaches based on classification algorithms [38], approaches based on artificial intelligence [26], [39] and an approach based on fuzzy-set-theory [40]; Empirical studies are typically performed by taxonomists or biologists on their own taxa to provide a reference dataset and to address issues such as phylogenetic relationships [41], [42], species assignments of unknowns [41], [43], and resolution of cryptic species complexes [44]–[46]. Until recently, relatively few barcoding studies have been performed to address ecological and evolutionary issues, such as species interactions, host-parasitoid relationships, food-web structures, DNA barcode accumulation curves [46]–[52]. In contrast, studies of microbiological community diversity have been more observant of the potential of diversity assessment with DNA barcoding [53]–[66]. However, the use of DNA barcoding for estimating biodiversity indices has not been widely evaluated for plants or animals [67], [68], although some insect groups have been so-studied, including ants [69], [70], flies [71]–[73], and wasps [74], [75].

Here we focus on Tibetan moth species (family Noctuidae) to evaluate community species diversity with a DNA barcoding (DB)-based method, and compare it to the traditional morphology (TM)-based method. The Noctuidae constitute one of the largest lepidopteran families, containing some 20,000 species from 20 subfamilies worldwide. A total of 3,751 species have been reported in China [76]–[80]. The larvae of many of these species are pests of agriculture and forestry, causing serious damage to agricultural production in China [81]. Tibet, known as the roof of the world, has an average altitude of 4,000 meters. Its unique natural environment has resulted in unique assemblages of species and particular morphological adaptations. For example, Tibetan noctuid moths resident above 4,000 meters have smaller body sizes, darker body color, and/or more setae, which are thought to be adaptations to the strong winds and low temperatures at high elevations [81]. The Tibetan Plateau is a biodiversity hotspot and contains many rare species [82]–[85]. However, owing to the lack of documentation and a largely undescribed species assemblage, biodiversity assessments of the region have generally omitted invertebrates, which probably make up majority of the region's diversity. Since species identification with different barcoding methods has been systematically evaluated elsewhere [39], [40], [86]–[90], our study has three aims: (i) to investigate the relationship between TM-based and DB-based measures of species diversity; (ii) to compare species diversities of noctuid moths across different environmental gradients on the Qinghai-Tibetan Plateau; and (iii) to examine relationships between environmental factors and species diversity for this group.

## Results

### Phylogenetic Analysis and DNA Barcode Gaps

**Figure 1:**
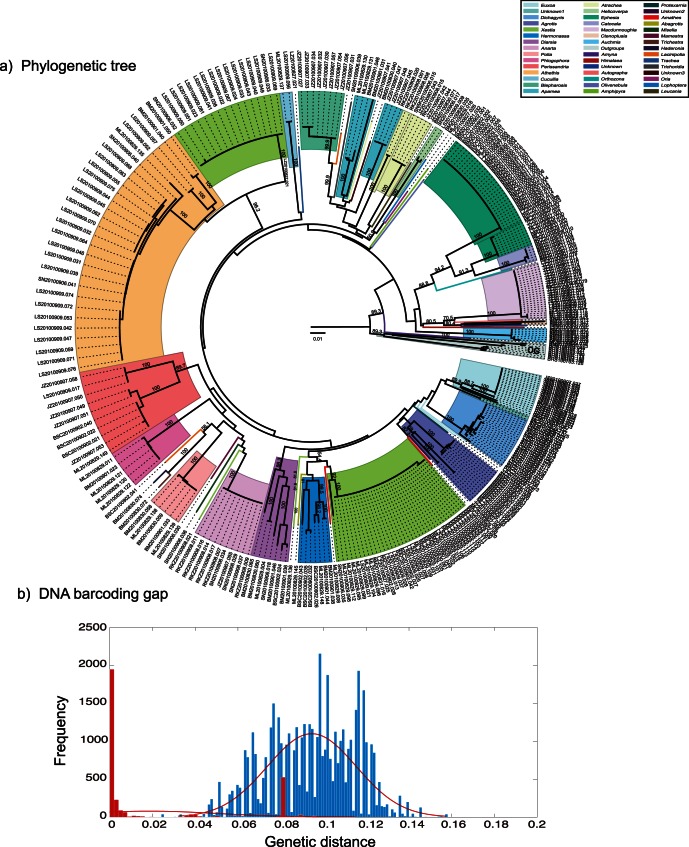
(a) Phylogenetic tree of Tibetan moth species from the family Noctuidae, inferred using neighbor-joining analysis of 320 COI DNA sequences. **Different colours represent different genera.** (b) Plot of genetic distances showing the barcoding gap. Intraspecies in red, interspecies in blue. Fitted normal distribution curves are shown in red.

Our 615-bp alignment comprised 328 *COI* sequences, sampled from 68 species and 45 genera. After morphological examination, ten species were considered as potential new species based on differences in genitalia. These were included in our calculations of diversity because, upon being identified as ten distinct new species, they would not affect the calculation of diversity. These ten potential new species could be named as spe1, spe2, spe3, 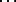, spe10, symbolically, and species diversity could be computed as they are considered known species; no mathematical complications arise from the potential new species identified here as distinct species. Twenty-two genera comprised just a single individual of a single species, and these singletons are considered to be rare species in the community. The remaining 23 genera were each represented by more than one individual, with most of these forming monophyletic clades at the species level. There were two exceptions, *Xestia* and *Apamea*; the former was split into two distant clades on the NJ tree, whereas the latter was split into two subclades by *Apamea* and two unassigned sequences which might belong to potential new species. The *COI* barcode showed an average interspecific K2P distance of 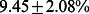, which is about five times the mean intraspecific distance (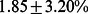). However, there was no clear DNA barcode gap for the *COI* barcode (Fig. 1), indicating the difficulty of distinguishing sibling species and/or potential cryptic species within this lepidopteran group. These results are generally consistent with those based on the phylogenetic tree. Of the 58 known morphologically identified species, 23 (39.7%) were endemic to the Qinghai-Tibetan Plateau. A comparison of the tree topology between the neighbor-joining tree and the maximum-likelihood tree is presented in Appendix S2. There are some inconsistencies between them, indicating that tree-based barcoding methods are not suitable for determining the phylogeny of our Noctuidae moths. However, it is to be expected that a robust tree topology is not easily achieved with a short DNA marker, like the 648 bp COI barcode region.

### Comparing Measures of Species Diversity

**Figure 2:**
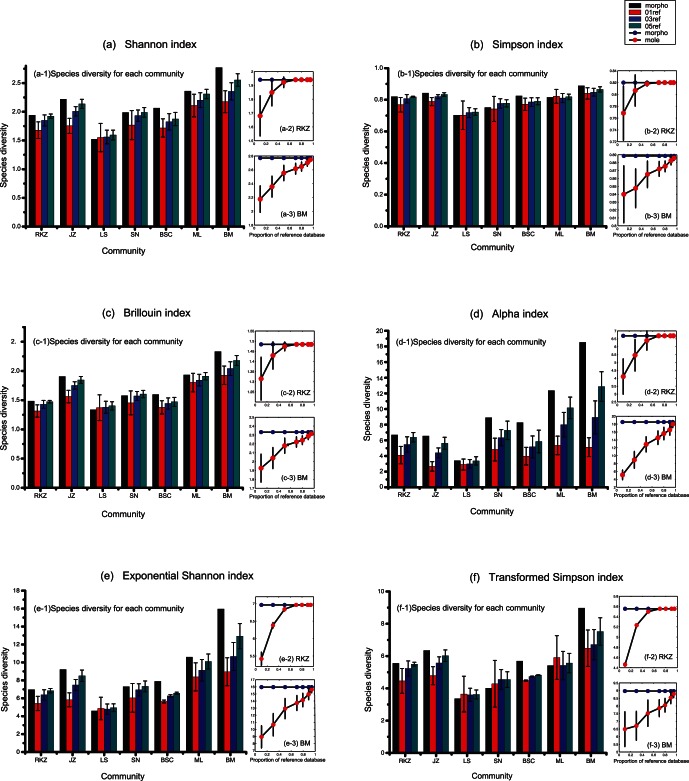
Species diversities of Tibetan moth species (Noctuidae) for each community, calculated using different diversity indices. (a) Shannon index; (b) Simpson index; (c) Brillouin index; (d) 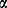 index; (e) Exponential Shannon index; (f) Transformed Simpson index.

We computed both TM- and DB-based diversities for each community using the six different indices (Fig. 2a–e). For each community, we tested the null hypothesis that DB-based measures of species diversity do not differ from TM-based measures using 1000 replications for each of the six diversity indices. We did not detect any significant differences between TM- and DB-based diversities for the 03 ref and 05 ref data sets, which represent 30% and 50% proportions of database reference sizes respectively (Fig.2, Appendix S2). For instance, the TM-based Shannon index for the Bome community was 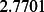, while the DB-based values were 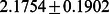
(01 ref), 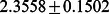
(03 ref), and 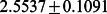
(05 ref) (Fig.2a; Appendix S2; 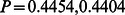, and 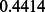 respectively). This indicates that DB-based measures of species diversity can serve as good surrogates for TM-based measures in most of the situations tested in this study. Even for smaller reference sizes (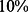), we also failed to reject the null hypothesis, with only a few exceptions such as the Shigatse, Lhasa, and Pagsum Co communities (Fig. 2a–f.; Appendix S2). Compared with TM-based species diversities, DB-based methods tended to slightly underestimate diversities. For example, the TM-based Simpson index of the Shigatse community was 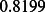, whereas the DB-based values were 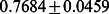
(01 ref) and 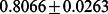
(03 ref) (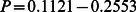; Fig.2a; Appendix S2). However, this could be largely corrected mathematically (see below). There were strong correlations between TM and DB-based diversities for these seven communities (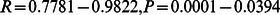; Appendix S3). Simulations with different reference library sizes indicated that the larger the proportion of the reference database for DB-based species diversity, the closer become these estimates of diversity to the TM-based species diversity (Fig. 2a–f; Appendix S4). For example, for a low proportion of reference database (10%; 01 ref), a diversity value of 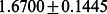 was estimated for the Shigatse (RKZ) community using the Shannon index and the DB-based method (Fig. 2a–2; Appendix S4). With an increasing proportion of the reference database (30% of reference database; 03 ref), the Shannon index for the Shigatse community increased (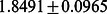; Fig.2a–2; Appendix S4), approaching the TM-based Shannon index value for the same community of 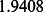 (Fig. 2a–2; Appendix S4). For the Bome (BM) community, the same tendency was observed (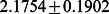, 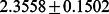,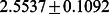, for 01 ref, 03 ref and 05 ref, respectively with DB-based diversity measurements; Fig. 2a–2; Appendix S4). The 05 ref value was close to the TM-based species diversity measure (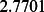; Fig. 2a–2; Appendix S4). As mentioned above, the other five diversity indices show the same pattern as the Shannon index (Fig. 2b–f; Appendix S4).

### Rank-abundance Plots and 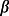 Diversity

**Figure 3:**
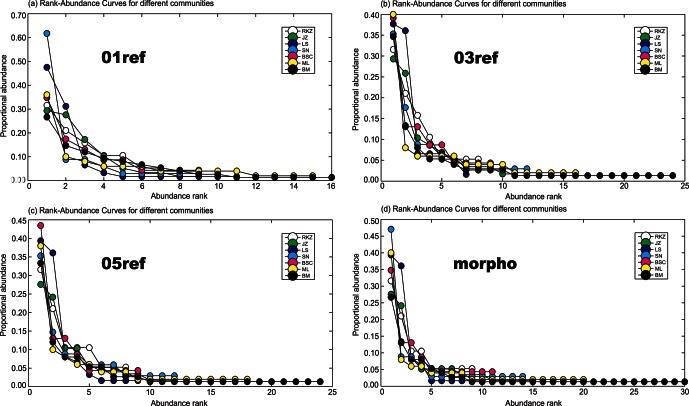
Rank-abundance curves of each community based on DNA barcoding (DB) with different reference database (a–c) or traditional morphology (TM) (d). (a) DB-based rank-abundance curves for each community based on reference size of 10% (01 ref). (b) DB-based rank-abundance curves for each community based on a reference size of 30% (03 ref). (c) DB-based rank-abundance curves for each community based on a reference size of 50% (05 ref). (d) TM-based rank-abundance curves for each community.

We obtained both TM-based and DB-based rank-abundance (RA) curves for each community in Tibet (Fig. 3a–d). For the latter, we further examined the effect of different reference library sizes on the estimation of RA curves. All DB-based RA curves gave similar and clear patterns for all seven communities when compared to the TM-based RA curve. For example, all DB-based RA curves showed that the Bome community in the east of Tibet has the highest species richness and species evenness (16,24, and 24 for 01 ref, 03 ref and 05 ref respectively), whereas the Shigatse community in the west of Tibet has the lowest species richness and species evenness (6, 8, and 8 for 01 ref, 03 ref and 05 ref respectively); the TM-based RA curves gave values of 30 for Bome and 9 for Shigatse (Fig. 3a–d). The DB-based RA curves appear to underestimate species richness for each community to some degree, depending on the reference database. Therefore, caution should be still exercised when using DB-based RA curves in an ecological investigation, especially if the reference library is small.

A Jaccard similarity index of 1 means that all species are shared across all areas, while an index of 0 means that no species are shared among the areas compared [8]. The average TM-based and DB-based Jaccard similarity indices were 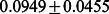 and 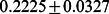, respectively, reflecting the low level of species sharing among the sampling sites. Mainling and Lhasa, Bome and Pagsum Co, had the highest values for the Jaccard similarity index (0.1724 and 0.1724, 5 species respectively), whereas Shigatse and Lhasa had the lowest Jaccard similarity index of 0.0217 (one species, Appendix S5).

The DB-based Jaccard similarity index gave much higher values than the TM-based one, suggesting that Jaccard similarity indices were overestimated by the DB-based method (Appendix S5). However, additional analyses with Mantel tests yielded significant correlations between TM- and DB-based Jaccard similarity indices (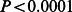, 1000 replications), indicating that TM-based and DB-based Jaccard similarity indices are in fact highly correlated.

### Variation in Diversity across Different Environmental Gradients on the Qinghai-Tibetan Plateau

**Figure 4:**
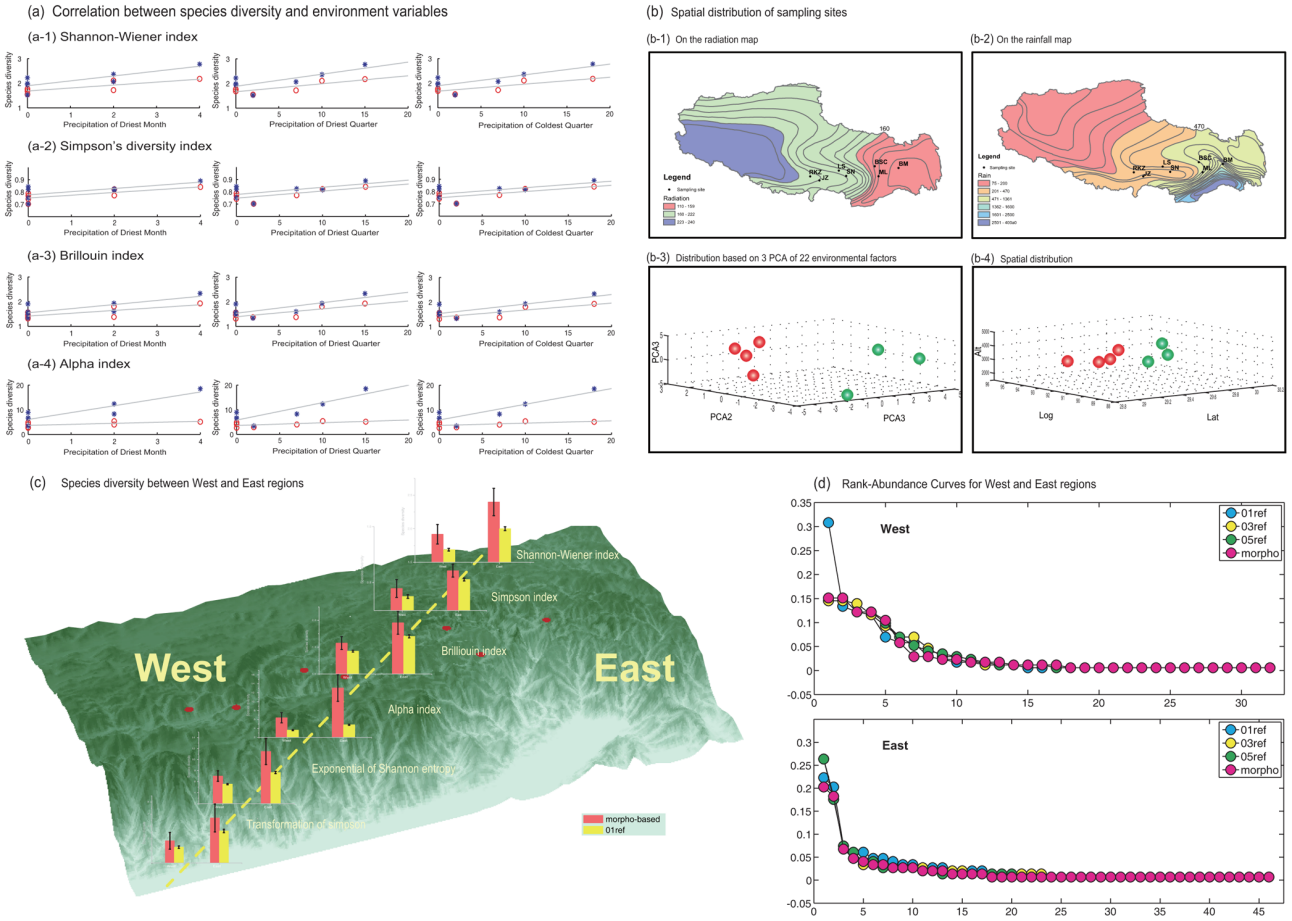
Correlations between species diversity and environmental factors. (a) Significant correlation between species diversity and three environmental factors, including precipitation of driest month, precipitation of driest quarter, and precipitation of coldest quarter. (b) Distribution of sampling sites/communities along different environmental gradients, including radiation and rainfall. Clustering of seven communities based on PCA of 22 environmental factors. (c) Spatial distribution of species diversities between West and East regions. (d) Rank-abundance curves of West and East communities.

Among 22 environmental factors, three (Precipitation of Driest Month, Precipitation of Driest Quarter, and Precipitation of Coldest Quarter) were found to be significantly correlated with species diversity. For example, results for the Shannon index were 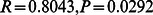 for TM-based species diversity and Precipitation of Driest Month, 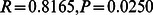 for DB-based species diversity and Precipitation of Driest Month; 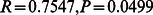 for TM-based species diversity and Precipitation of Driest Quarter, 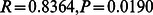 for DB-based species diversity and Precipitation of Driest Quarter; 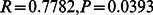 for TM-based species diversity and Precipitation of Coldest Quarter, 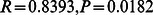 for DB-based species diversity and Precipitation of Coldest Quarter; for the Brillouin and 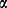 indices (see Fig. 4a). However the Simpson index gave weaker correlations between environmental factors and species diversities (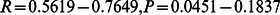 for both TM- and DB-based species diversities; Fig. 4a). It is clear that different diversity indices varied in their power to identify correlations between species diversities and environmental factors. The 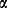 index yielded the strongest correlations, the Simpson index the weakest; the remaining indices showed intermediate values (Fig. 4a).

TM- and DB-based measures of species diversity differ slightly in their ability to detect correlations between ecological factors and species diversities, depending on which species-diversity measure was used. For the Shannon index, DB-based measures detected a greater number of correlations between environmental variables and species diversities than TM-based measures. For example, a significant negative correlation between species diversity and altitude was detected by DB-based but not TM-based measures of species diversity (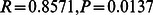 for DB-based;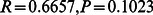 for TM-based; Fig. 4a).

Initial investigation of the spatial distribution of the seven communities, based on radiation and rainfall, found that they were distributed in two different ecological regions. In the west, the Shigatse, Gyantse, Lhasa, and Shannan communities are subject to strong radiation, low rainfall, and low humidity. In the east, the Pagsum Co, Mainling, and Bome communities are subject to weak radiation, high rainfall, and high humidity (Fig. 4b). Principal-components analysis based on all 22 environmental factors further confirmed that these seven communities belong to two different types of ecological environments: West and East (Fig. 4c; see also cover photos).

Further comparisons between these two regions indicated a significant, or nearly significant difference in species diversities, depending on which diversity measures were used (DB- or TM-based) (Fig. 4de). On average, five of the DB-based diversity indices (Shannon, Simpson, exponential of Shannon, transformed Simpson, and 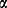), yielded higher values in eastern than western communities (Shannon, 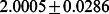 for the East and 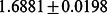 for the West, 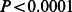; Simpson, 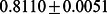 for the East and 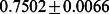 for the West, 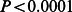; 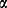, 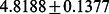 for the East and 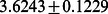 for the West, 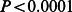; Fig. 4d.). The Brillouin index showed the same trend as the other five diversity indices, but without a significant difference (Brillouin indices of 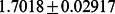 and 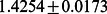 for the East and West communities, respectively; 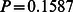; exponential of Shannon, 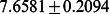 for the East and 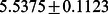 for the West, 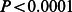; transformed Simpson, 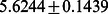 for the East and 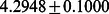 for the West, 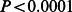; Fig. 4de). TM-based species-diversity analysis also generated a similar pattern to DB-based species diversity, showing a lower diversity in the West (Shannon, 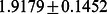; Simpson, 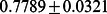; Brillouin, 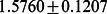; 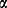, 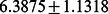; exponential of Shannon, 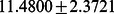; transformed Simpson, 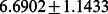; Fig. 4c) and higher diversity in the East (Shannon, 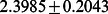; Simpson, 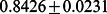; Brillouin, 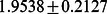; 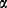, 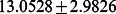; exponential of Shannon, 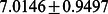; transformed Simpson, 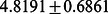; Fig. 4c), with nearly statistical significance (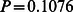; 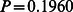). The close to significant result was also detected by the 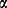 diversity index (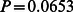; Fig. 4d), which has been shown to be the most sensitive diversity index by previous studies [1], [8] and in the present study.

## Materials and Methods

### Sampling, DNA Extraction, PCR and Sequencing

**Figure 5:**
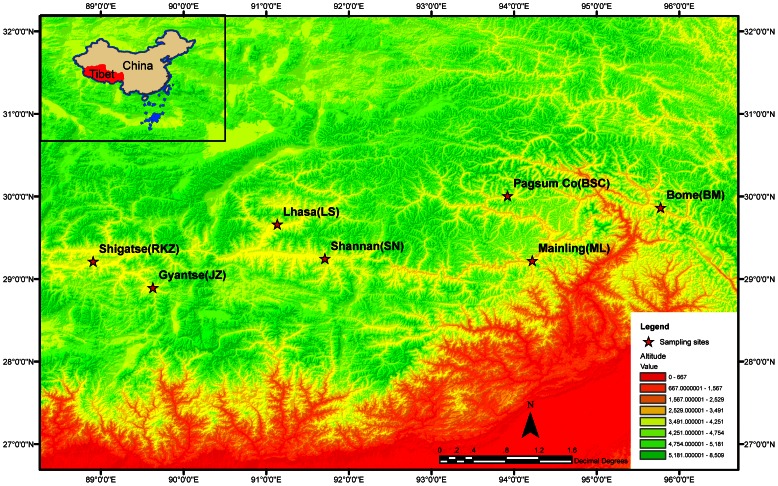
Sampling sites in the Qinghai-Tibetan Plateau in China. Samples were collected from seven locations from West to East in Tibet, representing different ecological conditions. RKZ - Shigatse, Gyantse - JZ, Lhasa - LS, Shannan - SN, Pagsum Co - BSC, Mainling - ML, and Bome - BM.

We sampled 320 specimens from species of the moth family Noctuidae from seven locations in Tibet (Fig. 5). Samplings were performed with traditional light trap methods collecting moths overnight, typically, in a relatively short time period of some two weeks (Aug. 26-Sept.11) of the year, to avoid the effect of seasons on the diversity assessments. These specimens represented 68 species and 45 genera (Appendix S6; no specific permits were required for the described field studies, the locations are not privately-owned or protected in any way, and the field studies did not involve endangered or protected species). Three species (eight specimens) from the family Pyralididae [91]were included as outgroup taxa when inferring phylogenetic trees. Specimens were identified by one of us, an expert lepidopterist in East Asia (H.L.H.) [81], [92]–[100]. Decisions on species status were based exclusively on morphological evidence, with male genital characters further examined when necessary (Appendix S7). DNA samples were prepared from individual insects by extraction of total DNA from animals either frozen or preserved in 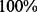 ethanol. Genomic DNA was extracted using a BIOMED DNeasy kit. The mitochondrial *COI* gene was amplified via PCR using rTaq (TAKARA) with the primers LCO1490 (GGTCA ACAAA TCATAA AGATA TTGG), and HCO2198 (TAAAC TTCAG GGTGA CCAAA AAATCA) [101]. The amplification reaction was performed in a total volume of 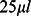, including 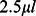 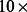 buffer, 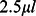
2.5 mM 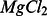, 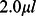 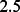 mM dNTP, 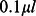 of each primer (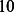 mM), 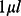 of template DNA, and 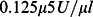 of DNA *Taq* polymerase, and 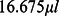 of distilled water. The PCR conditions were: 94°C for 2 minutes, 40 cycles of 94°C for 20 seconds, 54°C for 20 seconds, 72°C for 45 seconds, and a final extension at 72°C for 10 minutes. Sequencing was performed with an ABI3130 sequencer. The specimens were vouchered in a collection, and deposited in Capital Normal University (CNU, Beijing, China). Specimen data, trace files and sequences were deposited in BOLD in project Tibetan Lepidoptera (project code: NOCTU). All DNA sequences were also deposited in GenBank, with accession numbers JX392408 - JX392727.

### Phylogenetic Analysis

The raw DNA sequences were all checked manually and their ends were trimmed with BioEdit 7.0.1 (http://www.mbio.ncsu.edu/BioEdit/BioEdit.html). The resulting alignment of 615 bp contained no gaps and all sequences could be correctly translated into amino acids. To infer phylogenetic relationships among these species, we performed a neighbor-joining analysis [37] using MEGA 4.0 [102] with the K2P model of nucleotide substitution. We also analysed sequence alignments using maximum likelihood with the program PHYML3.0 [103]. A search using nearest-neighbor interchange was conducted to gain a preliminary estimate of the phylogeny. We then conducted a search using subtree pruning and regrafting to estimate the maximum-likelihood tree. The K2P substitution model was used [16], [17]. Branch support values were estimated using 1000 bootstrap replicates. All other parameters were set to their default values.

The distance between intraspecific and interspecific variation (the DNA barcode gap) is an important quantity in DNA barcoding practice. A large DNA barcode gap makes it easy to distinguish among species, whereas small or negative barcoding gaps tend to blur species boundaries and hamper species assignation. To explore intraspecific and interspecific variation in noctuid moths, we performed an analysis of DNA barcode gaps using a custom Perl script [41].

### Species Diversity Analysis

#### Traditional morphology-based method

Given the large number of indices, it is often difficult to decide which is the best method of measuring diversity [1], [3]–[8], [67]. In this study, we use the following criteria to choose diversity measures: discriminant ability, sensitivity to sample size, what component of diversity is being measured, and whether or not the index is widely used. Six diversity indices were selected: the Shannon index, the exponential of Shannon index, the Simpson index, the transformed Simpson index, the Brillouin index, and the log series, 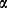 index [1], [3]–[8], [67].

The most widely used measures of diversity are the information theory indices, which treat the diversity in a natural system in a similar way to the information contained in a message. The Shannon index assumes that individuals/species are randomly sampled from an effectively infinite population or community [104]. It is calculated using the equation:
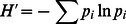
(1)The quantity 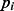 is the proportion of individuals found in the 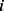
th species. The probability that any two individuals, randomly drawn from an infinitely large community, belong to different species is (Simpson 1949):
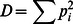
(2)where 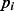 is the proportion of individuals in the 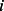
th species. As 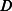 increases, diversity decreases and so the Simpson index is usually expressed as:



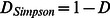
(3)The Shannon index and the Simpson index, which have moderate discriminant ability and moderate sensitivity to sample size, are commonly used in ecological studies [105]–[107]. However, some have recently recommended the use of the exponential of the Shannon index and the transformed Simpson index because they have the “doubling property [3]–[7]. The exponential of the Shannon index is given by.
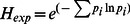
(4)and the transformed Simpson index is given by



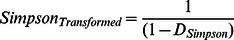
(5)The Brillouin index, which is not widely used, is given by.
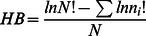
(6)where 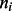 is the number of individuals of species 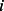 and 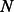 is the total number of individuals in the sample. We also used the log series index, 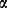, owing to its good discriminant ability and because it is not unduly influenced by sample size [1], [8], [67]. The log series takes the form:
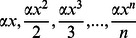
(7)where 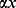 and 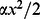 is the number of species predicted to have one and two individuals respectively, and so on (Fisher et al. 1943; Poole, 1974). The index can be obtained from the equation:
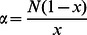
(8)where 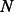 is the total number of individuals, 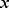 is estimated from the iterative solution of
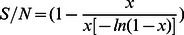
(9)


#### DNA barcoding-based method

**Figure 6:**
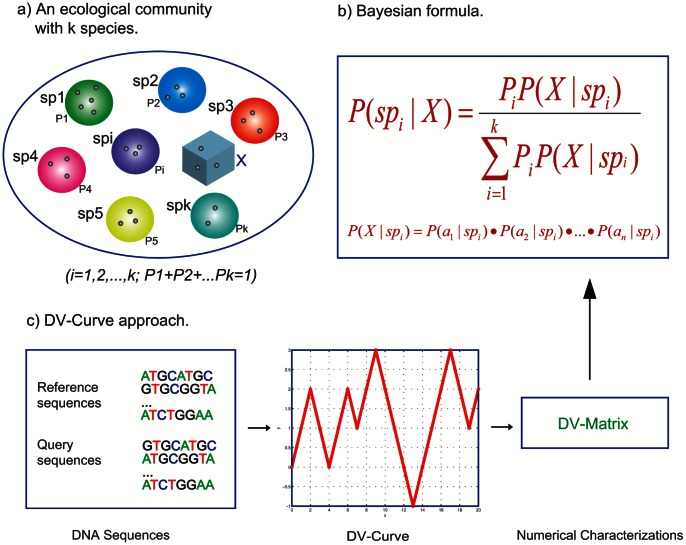
Species identity inferred via DNA barcoding for a community with a Bayesian method. (a) An ecological community with species. (b) Bayesian formula used to infer species membership for unknown samples. (c) DV-Curve approach to construct DV-Matrix for Bayesian inference. 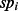 is the 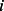
th species in the community, 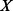 is an unknown sample with DNA sequenced, 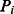 is the probability of a sample belonging to 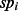. 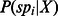 is the conditional probability of the unknown sample 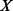 belonging to 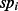 given that its DNA is sequenced. 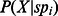 is the probability of having DNA sequence of the unknown sample 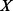 given that DNA sequences of 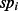 are known.

For a community with a very large number of hyperdiverse taxa, such as arthropods, investigations of species diversity are usually hindered by the difficulty of identifying species by traditional morphological means alone. DNA-based species identification [16], [17] provides an alternative method for assessment of species diversity. The commonly used barcoding gene, *COI*, was sequenced for all samples. Their species identities were assessed by matching their *COI* barcodes against a reference barcode library. Generally, species in a community are not necessarily closely phylogenetically related, but they are ecologically related, due to the broad taxon coverage and filtering of environmental factors for a community. Therefore, instead of using phylogeny-based DNA barcoding methods, a non-tree-based Bayesian approach, which takes advantages of both Bayesian theory and bioinformatics, was used to infer species identity via *COI* barcoding (Fig. 6).

The dual-vector curve (DV-Curve) was proposed by Zhang [108] as a two-dimensional graphical representation for visualizing and analysing DNA sequences (Fig. 6). It is able to represent DNA sequences without degeneracy and loss of information. Let us consider a DNA sequence 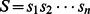 consisting of *n* nucleotide sites. Let 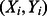 be the point of the DV-Curve, where 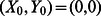 is the start point. The DV-Curve is uniquely determined by the following formula [108]:
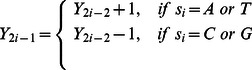
(10)

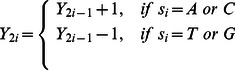
(11)

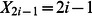
(12)

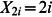
(13)where 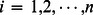.

To numerically characterize a DNA sequence via the DV-Curve, a 24-component vector 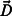 as described by Zhang [108] was used:
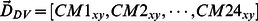
(14)


The 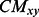 value [109] is calculated as follows:
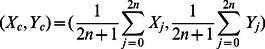
(15)

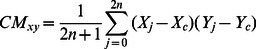
(16)


To get equation (14), we need to assign 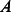, 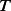, 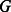, and 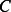 to basic Dual-Vectors in 4! different ways to have 4! = 24 different DV-Curves for a given DNA sequence. The vector 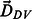 is further used as the input for the Bayes prior attributes.
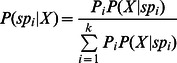
(17)Where 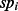 is the 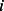
th species in the community, 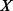 is an unknown sample with a DNA sequence, 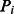 is the probability of a sample belonging to 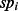. 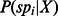 is the conditional probability of the unknown sample 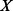 belonging to 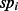 given that its DNA is sequenced. 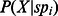 is the probability of having the DNA sequence of the unknown sample 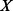 given that the DNA sequences of 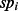 are known (Fig.6).

To explore the effect of reference library size on the assessment of species diversity, we randomly selected as the reference library different scales of the reference database, including 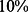, 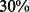, 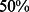, 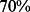, 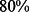, 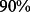, and 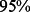 of the whole dataset. These are subsequently referred to as 01 ref, 03 ref, and so on. The full reference library comprises the 322 barcoded and morphologically identified specimens. Once species identities were inferred with barcoding-based methods, species diversity indices were calculated following equations 1–6 and 9. We computed species diversity for each of seven communities in Tibet, and for the whole Tibetan community. All of these calculations were performed with 30 random replications. The differences in species diversity among different communities were statistically examined via a permutation test with 1000 replications. Likewise, the permutation test was also applied to assess the differences in species diversities between morphology-based and barcoding-based methods. To investigate further the relationship between TM- and DB-based species diversities, we additionally performed a correlation analysis between these two types of measurement for all six diversity indices.

### Rank-abundance Curves Based on Morphology or DNA Barcodes

Species abundance distribution can be visualized in different ways, of which the rank-abundance curve (rank/abundance plot) is one of the best known and most informative ones [67], [110]. In this species are plotted in sequence from most to least abundant along the horizontal axis, with abundances displayed on the y axis [67]. One of the advantages of a rank-abundance curve is the clearly display of contrasting patterns of species richness and their relative abundances, compared with the inefficient presentation of a histogram [67], [111].

By plotting the relative abundance of species against their rank in abundance, we can readily gain information about the diversity of species within a community. We investigated whether DB-based rank-abundance curves could serve as an effective surrogate for TM-based rank-abundance ones. Therefore, we computed both TM- and DB-based rank-abundance plots, and further examined them with the Kolmogorov-Smirnov two-sample test [112].

### Beta Diversity and Correlations between Species Diversities and Environmental Factors

Diversities (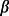) between sites/communities were compared using the Jaccard Index of similarity [8], 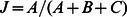, where A is the number of species shared between the two sites, and B and C are the number of species unique to each site.

To explore the relationship between species diversity and environmental factors, we examined 22 variables, including annual mean temperatures, mean diurnal range, and precipitation of the driest quarter (Appendix S8). Climate data are from the WorldClim dataset [113]. Both TM- and DNA-based indices of species diversity were calculated for the six species-diversity indices. Correlation analyses were performed between each species diversity index and each environmental factor.

The seven sampling sites/communities are distributed across quite different ecological conditions in Tibet, ranging from the dry west to the wetter east. We conducted a principal-component analysis [114] of the 22 environmental variables in order to reduce the correlation between these variables and to cluster the seven sites/communities.

Diversities of moth species between these two regions (Shigatse, Gyantse, Lhasa, and Shannan in the west, and the remaining three communities in the east) were further compared for the six diversity indices using a t-test. We also constructed rank-abundance plots and evaluated them with the Kolmogorov-Smirnov two-sample test for the two regions [112].

## Discussion and Conclusions

In our study of biodiversity assessment via DNA barcoding for a Tibetan moth community, we found that diversity measurements based on DNA barcoding are able to serve as good surrogates for morphology-based measures. Compared with traditional morphology-based methods, a DNA barcode approach, along with appropriate analytical procedures as outlined here, is fast and convenient and can greatly reduce the time needed for specimen identification. However, we note that our current method is subject to some limitations, for example, requiring a pre-defined reference library.

We have not applied a method based solely on molecular operational taxonomic units, although such approaches have been successfully implemented elsewhere. One of the first ecologically-targeted barcoding studies, of Madagascan ants, applied COI distance thresholds of 2% and 3% to differentiate taxa; it showed that biodiversity richness was not significantly different whether estimated by morphological means or from molecularly-defined taxa [70]. Microbiological community studies have applied a threshold of 3% dissimilarity in 16 S rRNA sequences to identify conspecifics [54], [56], [60], [61]. In fact, barcoding has proven to be an excellent tool for biodiversity surveys where the studied taxa do not have a solid taxonomic foundation [115].

However, an arbitrary threshold may be largely taxon-dependent [24], [28], [116], and was not applied in current study. Besides the threshold method, the generalized mixed Yule-coalescent (GMYC) model could be an alternaltive approach for species delimitation based on DNA sequences [21]. The GMYC method models branching events between species with a Yule model [117] and branch events within species using a neutral coalescent model [118]. The method has potential for biodiversity assessments although empirical studies are desirable to examine its consistency with traditional morphspecies since splitting or lumping was often observed compared to traditional taxonomy. An artificial intelligence-based approach, such as BP-based species identification [26], could be another choice for species identification, but was not applied to the current study due to its relatively slow training process during the construction of neural networks, making it impractical for the simulation study. The development of fast algorithms of this method for large datasets is highly desirable. In addition, it is generally recognised that multiple markers, especially the inclusion of nuclear genes, would increase the accuracy of species delimitations, and therefore the accuracy of species diversity assessment. Howver, we only applied the standard COI barcode in assessments of species diversity because of the widespread generality of the commonly used primers [16], [101].

We applied a non-tree based Bayesian method for species identification before the calculation of diversity mainly because species in a community are not necessarily closely phylogenetically related, although they are ecologically related, due to the broad taxon coverage and filtering of environmental factors for a community. Tree-based methods, including Bayesian phylogenetic methods, are not always able to produce reliable assignments of specimens when taxon sampling is incomplete, as is the case here. In this study, we focus mainly on assessments of species diversity, not barcoding approaches, as the latter have been systematically evaluated with both simulated and empirical data [39], [40], [86]–[90] and are outside the scope of the present study.

We found a significant positive correlation between species diversity and precipitation of driest month/coldest quarter for this taxon group. This environmental factor might affect the survival of pupae by changing the moisture of the micro-environments, or by influencing the diversity of their host plants. Our results are supported by the significantly higher diversity in the drier eastern communities compared with the wetter western communities on the Tibetan Plateau, where dramatically different ecological landscapes are present. However, owing to the lack of historical climate records in the sampling sites under investigation, the climate elements considered, such as monthly precipitation and mean, minimum, and maximum temperature, were all derived from the WorldClim dataset [113], which has been commonly used in ecological studies, but is restricted to records from the 1950–2000 period. However, this is unlikely to significantly affect our basic conclusions, because the assemblage of species for a certain community is not a consequence of short time interactions between species and environments, and among species but a long-term adaption to certain habitats.

In the future, biodiversity assessments may be further accelerated by meta-barcoding [119]; meta-barcoding of bulked arthropod samples has recently been shown to provide good estimates of within and between community diversity [120]. Whether the preference is for the sequencing of bulked or individual specimens, it is abundantly clear that DNA barcode-based biodiversity assessments are very effective and efficient alternatives to more time-consuming morphological-based measures that require considerable taxonomic expertise.

### Data Accessibility

DNA sequences: Genbank accessions JX392408 - JX392727.

Specimen data, trace files and sequences were deposited at BOLD under a project Tibetan Lepidoptera.

(Project Code: NOCTU; http://www.barcodinglife.com/index.php/MAS_Management_ProjectList).

## Supporting Information

Appendix S1 Comparison of tree topology between NJ and ML trees. The nodes in green on the tree indicate shared nodes between two trees, nodes in dark grey are nodes present on the NJ tree, but not on the ML tree.
(PDF)

Appendix S2 Permutation tests for TM and DB-based species diverisities, and diversities for each community.
(TXT)

Appendix S3 Correlation analysis between TM and DB-based species diversities for six different diversity indices.
(XLS)

Appendix S4 The effect of different reference database sizes on the estimation of species diversity.
(PDF)

Appendix S5

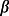
 diversity among different communities.(TXT)

Appendix S6 Sample localities in Tibet, geographical coordinates and sequences used for Noctuidae moth species.
(PDF)

Appendix S7 Species identification based on traditional morphology. a). Some pictures of moths used in this study; b). Female genitalia, example 1; c). Male genitalia, example 2.
(JPG)

Appendix S8 Twenty two environmental variables used in this study.
(TXT)
