# Supplementary material for: Quantifying Species Diversity with a DNA Barcoding-Based Method: Tibetan Moth Species (Noctuidae) on the Qinghai-Tibetan Plateau
Source: PLoS One. 2013 May 31;8(5):e64428. doi: 10.1371/journal.pone.0064428 (PMC3669328; doi:10.1371/journal.pone.0064428)

Appendix S1. Comparison of tree topology between NJ and ML trees.
The nodes in green on the tree indicate shared nodes between two trees,
in dark grey mean nodes present on NJ tree, but not on the ML tree.

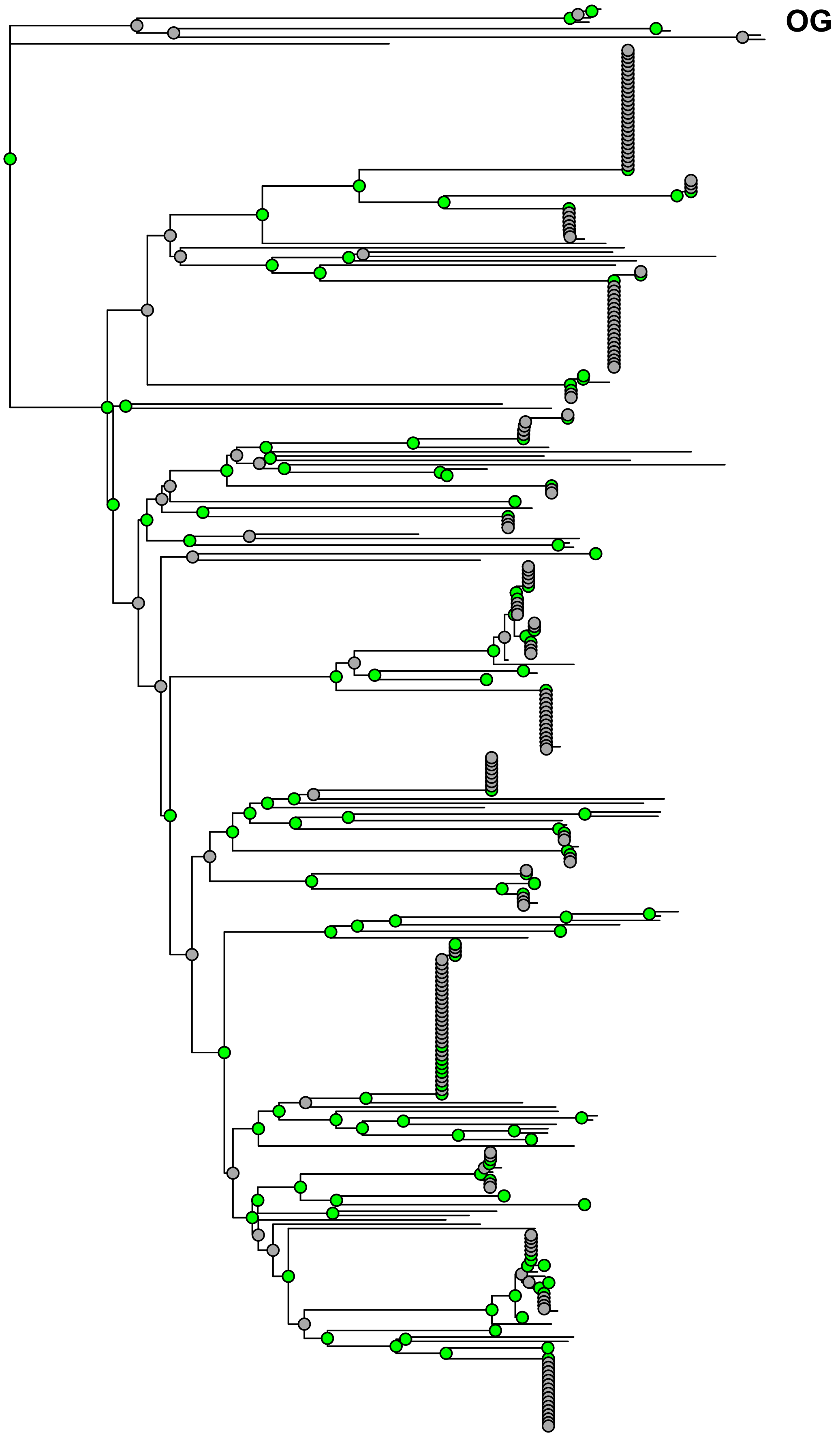

Supplement: Appendix S1 — Comparison of tree topology between NJ and ML trees. The nodes in green on the tree indicate shared nodes between two trees, nodes in dark grey are nodes present on the NJ tree, but not on the ML tree. (PDF) [file pone.0064428.s001.pdf]
