# Supplementary material for: Quantifying Species Diversity with a DNA Barcoding-Based Method: Tibetan Moth Species (Noctuidae) on the Qinghai-Tibetan Plateau
Source: PLoS One. 2013 May 31;8(5):e64428. doi: 10.1371/journal.pone.0064428 (PMC3669328; doi:10.1371/journal.pone.0064428)

Appendix S4 The effect of different reference sizes on the estimation of species diversity.

S4-1 Shannon-Wiener index

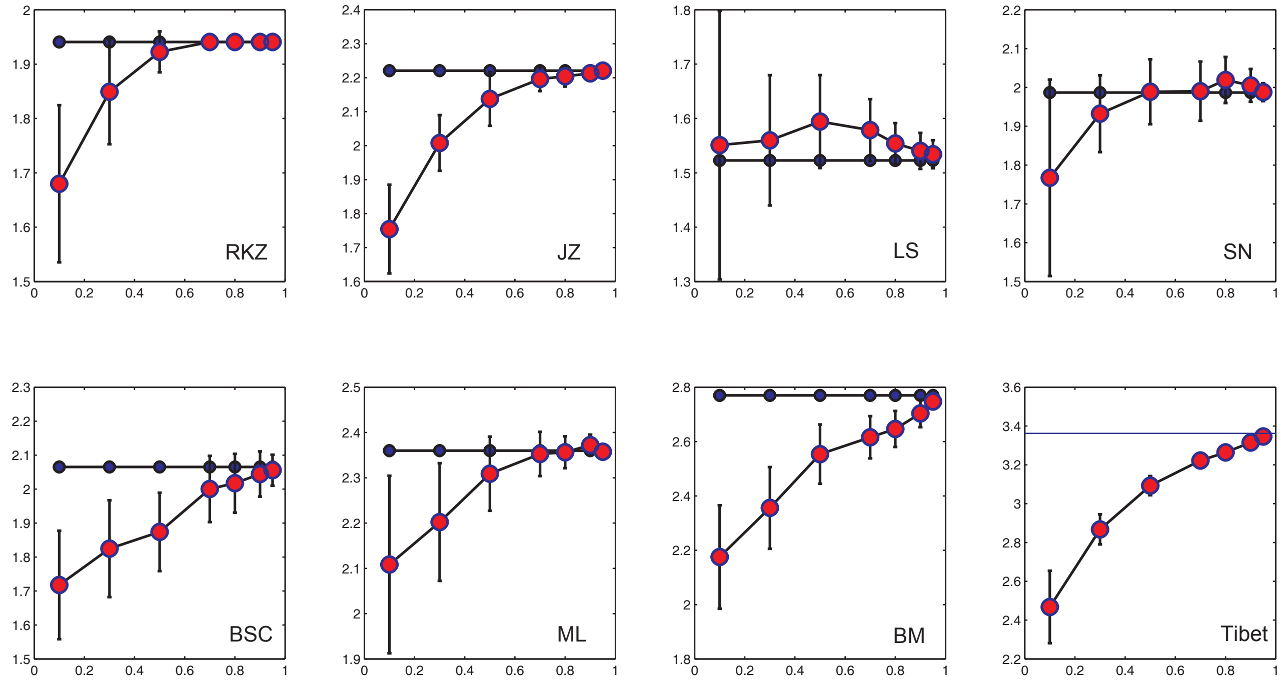

S4-2 Simpson index

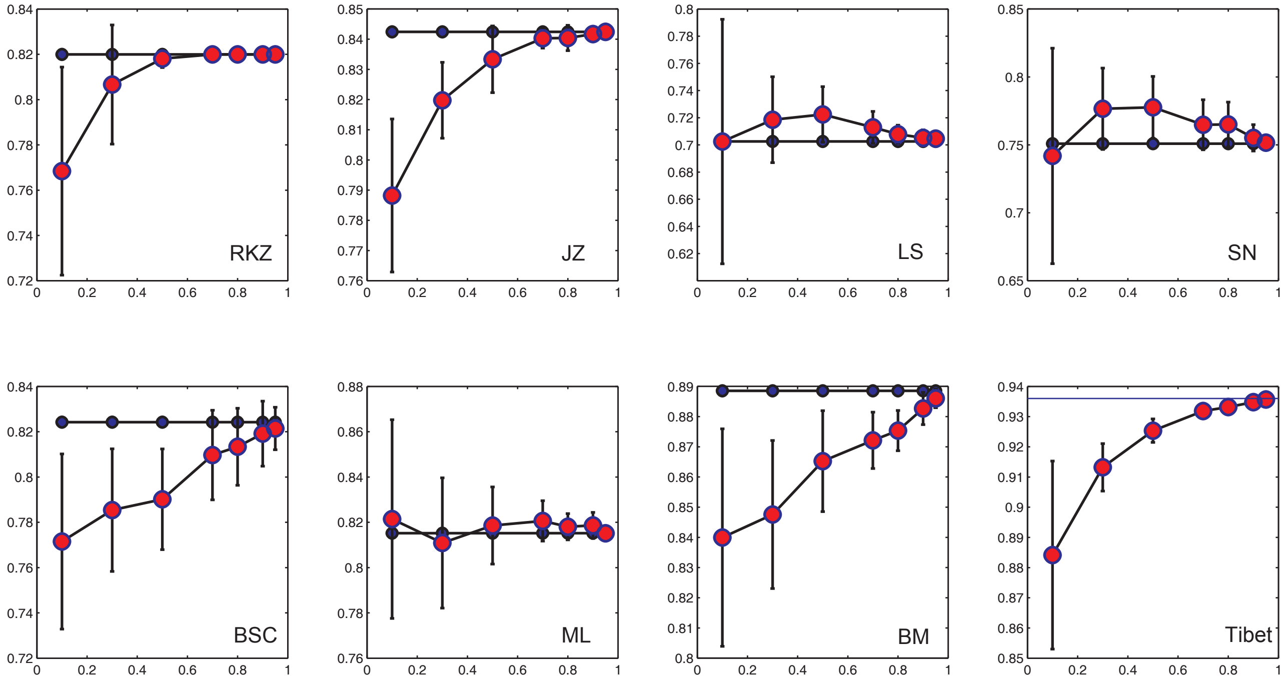

Appendix S4 The effect of different reference sizes on the estimation of species diversity.

S4-3 Brillouin index

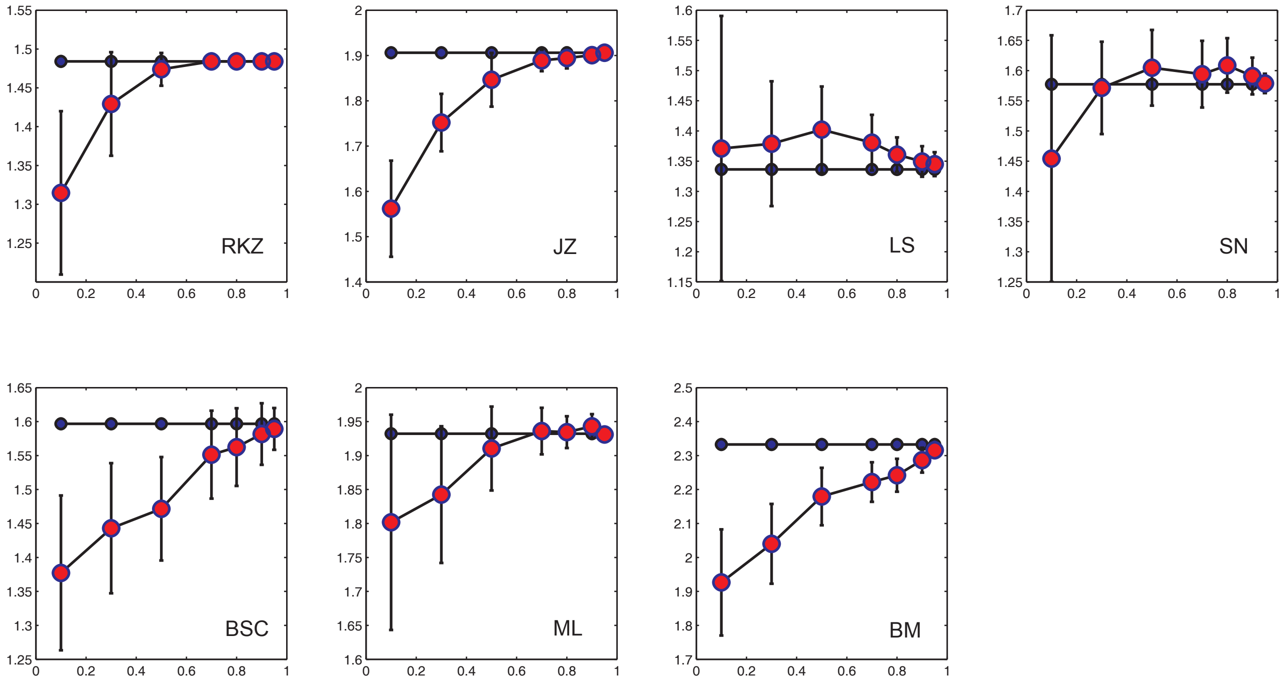

S4-4 Alpha index

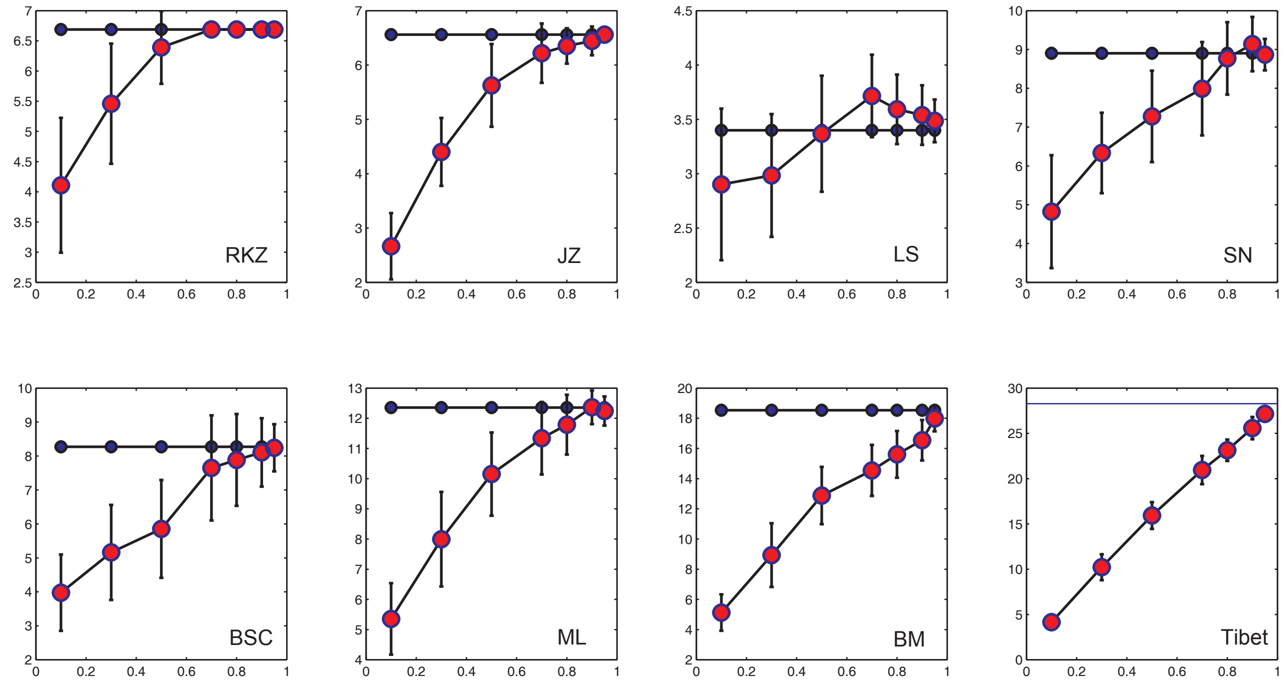

Supplement: Appendix S4 — The effect of different reference database sizes on the estimation of species diversity. (PDF) [file pone.0064428.s004.pdf]
