# Supplementary material for: Quantifying Species Diversity with a DNA Barcoding-Based Method: Tibetan Moth Species (Noctuidae) on the Qinghai-Tibetan Plateau
Source: PLoS One. 2013 May 31;8(5):e64428. doi: 10.1371/journal.pone.0064428 (PMC3669328; doi:10.1371/journal.pone.0064428)
Supplement: Appendix S6 — Sample localities in Tibet, geographical coordinates and sequences used for Noctuidae moth species. (PDF) [file pone.0064428.s006.pdf]

Appendix S6. Species identification based on traditional morphology.
a). Part of pictures of moths used in this study;
b). Female genitalia, example 1; c). Male genitalia, example 2.

(a) Pictures of moths

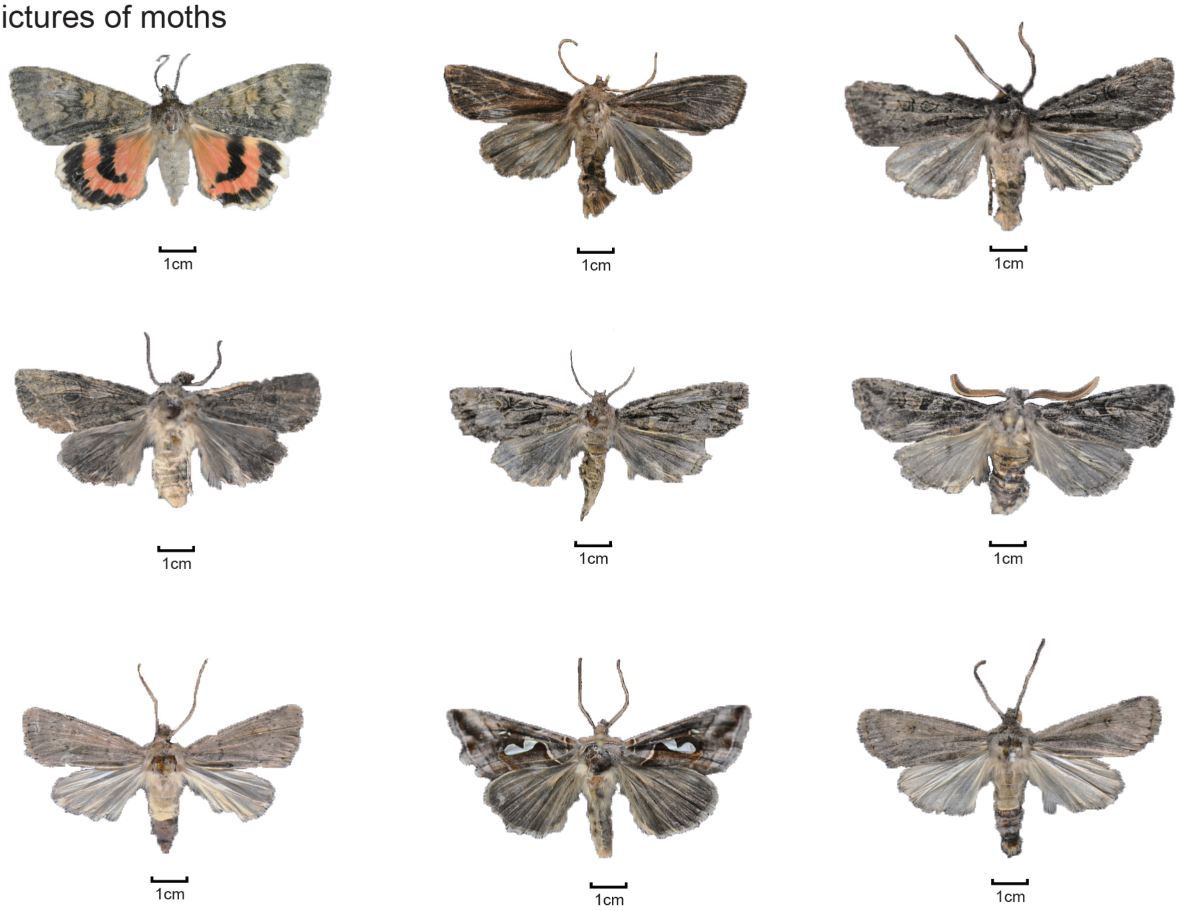

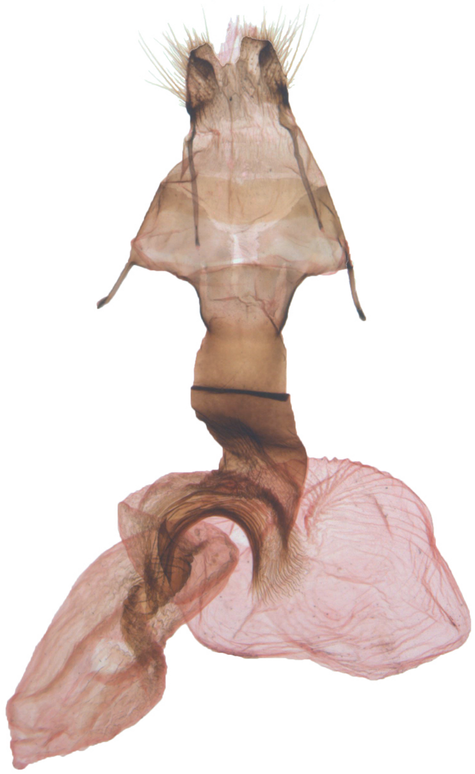

(b) Female genitalia

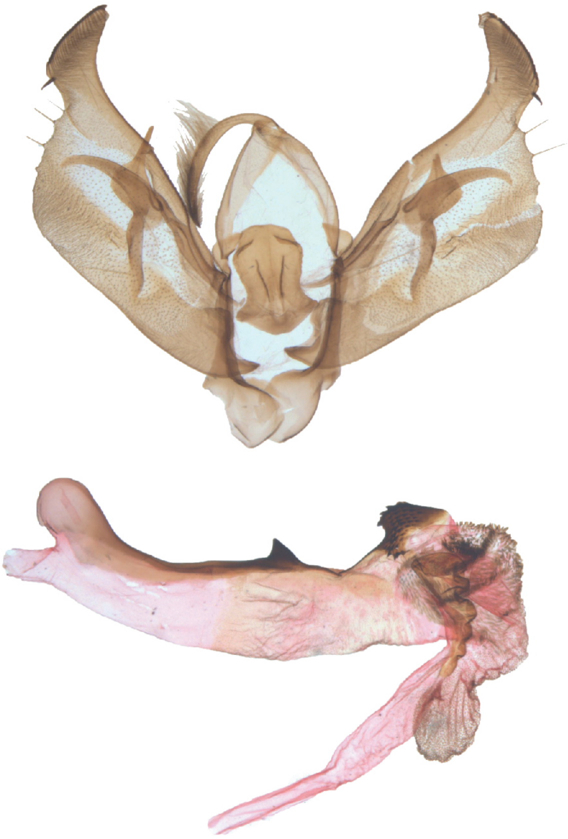

(c) Male genitalia
